# Increased concentrations of procalcitonin in patients with paracetamol intoxication

**DOI:** 10.1515/almed-2020-0108

**Published:** 2021-01-08

**Authors:** Luis García de Guadiana Romualdo, Carlos Rodríguez Rojas, Verónica Ramos Arenas, Rubén Cárdenas Gámez, María Dolores López Abellán, Mercedes González Morales

**Affiliations:** Laboratory Medicine Department, Santa Lucía University Hospital, Cartagena, Spain

**Keywords:** intoxication, paracetamol, procalcitonin

## Abstract

**Objectives:**

Paracetamol intoxication is one of the causes of elevated procalcitonin concentrations unrelated to infection. We report a case series of two patients intoxicated with paracetamol whose laboratory data revealed a significant elevation of serum procalcitonin concentrations without clinical, radiological and/or biological evidence of infection. The underlying mechanism by which paracetamol triggers an increase in procalcitonin concentrations is still unclear.

**Case presentation:**

We report two cases of paracetamol intoxication. Both patients were admitted to the Emergency Department (ED) and subsequently transferred to the Intensive Care Unit (ICU). The patients exhibited elevated procalcitonin levels during the first hours of admission without clinical and/or microbiological evidence of infection that could explain such increase. Notably, only Case 1 developed liver injury, with alterations in alanine aminotransferase (ALT), aspartate aminotransferase (AST), bilirubin and esterified bilirubin concentrations, which were not observed in Case 2.

**Conclusions:**

The two patients showed elevated procalcitonin concentrations resulting from paracetamol intoxication, although only a patient exhibited signs of liver injury. These findings suggest that increased procalcitonin levels induced by a paracetamol overdose cannot be fully explained by hepatocyte injury alone, but other mechanisms involving other organs and tissues may also be associated. In any case, although this mechanism is not well understood, it is important to be aware of this limitation when using procalcitonin as a biomarker of infection in patients intoxicated with paracetamol.

## Introduction

Procalcitonin is widely used as a biomarker for the diagnosis of severe bacterial infection and follow-up of response to antimicrobial therapy [1], [2]. Procalcitonin concentrations have been reported to be elevated in patients with C-cell carcinoma, burns, trauma [3] or intoxication with drugs such as amphetamines [4] or paracetamol [5]. In the latter case, liver cell injury cannot fully explain the elevation of procalcitonin concentrations, and other organs might be involved in the underlying mechanism of this phenomenon [6], [7], [8].

We report a case series of two patients intoxicated with paracetamol admitted to the Emergency Department (ED) who showed elevated procalcitonin concentrations without clinical or microbiological evidence of bacterial infection. Case 1 exhibited significantly elevated levels of hepatic function markers, whereas these markers were not altered in Case 2. This difference suggests that procalcitonin elevation does not only result from paracetamol-induced liver injury, but other mechanisms seem to be involved [5].

## Case presentation

### Case 1

A 65-year-old patient was admitted to the ICU with a decreased consciousness state after the ingestion of bromazepam, zolpidem and alcohol to an unknown amount 6 h before with the purpose of autolysis. The clinical history of the patient included chronic alcoholism and two episodes of common flutter that required ablation of the cavo-tricuspid isthmus and anticoagulation with acenocumarol and apiroxaban. On ED admission, vital signs were: blood pressure: 163/94 mm Hg; heart rate: 66 beats per minute; respiratory rate: 19 breaths per minute; temperature: 36 °C; and Glasgow Coma scale: 15. On admission, analytical results showed increased ethanol levels in blood and a negative urine drug abuse test (including benzodiazepines), without other significant findings (Table 1). Treatment with flumazenil was started, with a significant improvement of sensory capacity. After an interview with the Psychiatry Department, the patient reported having taken approximately 20 g of paracetamol. Paracetamol concentration in the first sample of blood was 162.4 μg/mL. N-acetyl cysteine therapy was maintained for 72 h, without significant alterations in hepatic function markers (ALT, AST and bilirubin). In the light of previous cases of procalcitonin elevation in patients with paracetamol intoxication, serum procalcitonin levels were measured without significant alterations.

**Table 1:** Laboratory findings in Case 1.

	Reference interval	ED admission Day 1	Time_12h_	Time_18h_	Time_24h_ ICU admission Day 2	Time_48h_ Day 3	Time_72h_ Day 4	Time_96h_ Day 5	Time_168h_ ICU discharge Day 6	Time_240h_ Day 8	Time_264h_ Day 9	Time_336h_ Day 12 Exitus
**Paracetamol, μg/mL**	<5^a^	162.4	51.8	18.6	6.3	–	–	–	–			
**ALT, U/L**	<41	31	170	319	472	6.988	4.386	2.643	438	210	143	139
**AST, U/L**	<40	35	248	449	691	11.939	2.277	1.342	59	60	55	–
**Bilirubin, mg/dL**	<1.2	0.4	3.2	3.8	3.8	3.7	4.1	4.3	1.9	1.7	2.2	1.2
**Direct bilirubin, mg/dL**	<0.3	–	2.3	1.9	2.7	1.9	3.3	3.5	1.7	1.3	1.5	–
**PT (ratio)**	0.9–1.1	–	1.54	2.24	2.53	3.24	3.24	1.81	1.32	–	1.22	1.7
**aPTT (ratio)**	0.9–1.1	–	0.97	1.00	1.03	1.01	1.01	–	1.01	–	0.83	0.84
**Procalcitonin, ng/mL**	–	0.04	23.15	20.61	21.69	11.84	6.18	4.04	1.97	0.69	0.60	2.45
**CRP, mg/dL**	<0.5	<0.03	<0.03	0.6	1.0	3.5	5.8	7.4	14.3	18.7	24.3	14.18

^a^Toxic concentrations after overdose: •4 h after overdose: >200. •8 h after overdose: >100. •12 h after overdose: >50. ED, emergency department; ICU, intensive care unit; ALT, alanine aminotransferase; AST, aspartate aminotransferase; PT, prothrombin time; aPTT, activated partial thromboplastin time; CRP, C-reactive protein.

Control laboratory tests performed 12 and 18 h from ED admission demonstrated liver dysfunction, with progressively increased levels of aminotransferases, bilirubin, esterified bilirubin, and prothrombin time, which required admission to the Intensive Care Unit (ICU). On ICU admission, paracetamol concentrations were 6.3 μg/mL and the patient showed biochemical alterations, suggestive of exacerbation of liver dysfunction. Blood samples collected during the first 24 h of admission showed a significant elevation of procalcitonin concentrations, although the patient did not exhibit any signs and/or symptoms of infection.

During ICU admission, the patient had an episode of atrial fibrillation that was reverted with amiodarone, and a bradycardia-tachycardia syndrome that required the implantation of a definitive pacemaker. The evolution of the patient at respiratory and neurological level was favorable, without signs and/or symptoms of infection, but with elevated levels of procalcitonin that decreased progressively. After 7 days in the ICU, the patient was discharged to the Internal Medicine Department, with a significant improvement of hepatic function markers.

**Figure 1:**
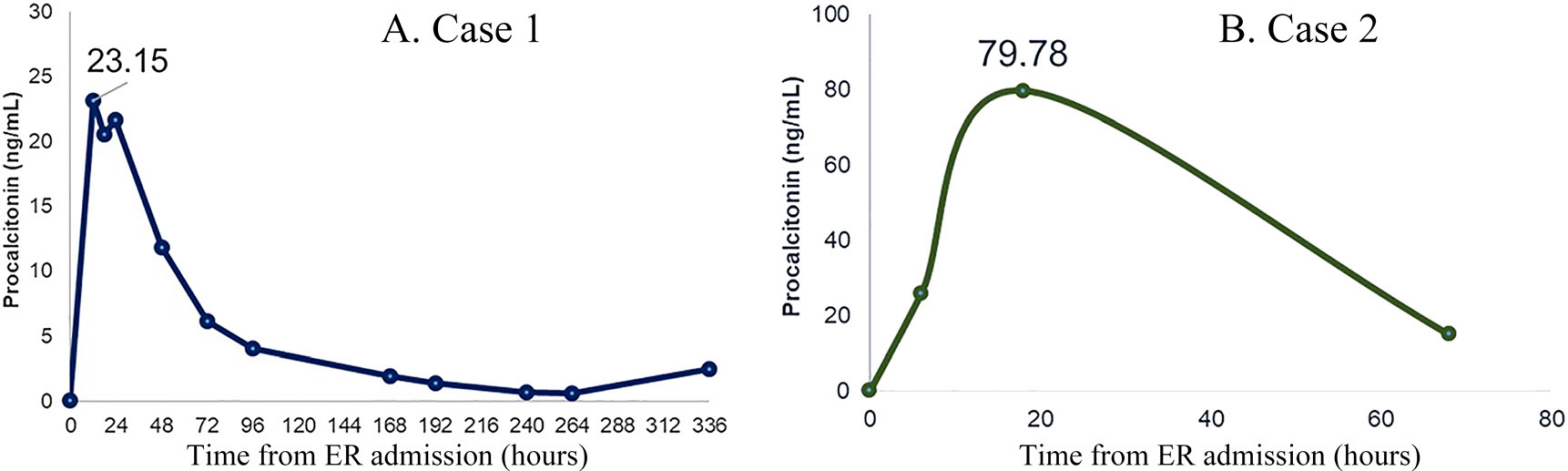
Kinetics of procalcitonin levels in the two patients. ER, Emergency room.

On admission to Internal Medicine Department, the patient showed a good initial general condition. However, the patient had an episode of paroxysmal atrial fibrillation with radiological worsening, which improved after the administration of furosemide. After amiodarone therapy was resumed, the patient developed a bilateral pulmonary edema. All microbiological tests were negative (PCR for SARS-CoV-2 and type A/B influenza virus, and urine *Legionella* and *Streptococcus pneumoniae* antigen). Biochemistry showed a progressive increase of C-reactive protein (CRP), which started in the ICU, and creatine kinase, which reached 7,434 U/L the day of exitus, when procalcitonin levels also increased significantly, after a progressive decrease (Figure 1A). The patient died after a hospital stay of 12 days from a non-cardiogenic pulmonary edema of unknown origin that could be related to hospital-acquired pneumonia, which was not confirmed microbiologically, or a pneumonitis associated with pulmonary amiodarone toxicity.

### Case 2

A 34 year-old patient admitted to the ED after a suicide attempt by drug intoxication with lorazepam (12 mg), trazodone (2 g) and paracetamol (38 g) that had occurred 12 h before. On admission, the patient had normal blood pressure, was afebrile, eupneic and disoriented, with a Glasgow Coma scale of 14.

After a gastric lavage and the start of N-acetyl cysteine therapy, the most remarkable analytical findings (Table 2) were high paracetamol concentrations and a positive urine benzodiazepine test result, without laboratory data of liver injury. Treatment with flumazenil was started at ICU admission, 18 h after the massive ingestion of drugs; N-acetyl cysteine therapy was maintained for 24 h more. Close clinical, electrocardiographic and biochemical monitoring was performed. During ICU stay, the patient remained clinically stable, without biochemical data of organ dysfunction, but with significantly elevated procalcitonin concentrations (Figure 1B) unrelated to infection. After 3 days in the ICU, the patient was discharged to the Internal Medicine Department with a favorable evolution. The patient was finally discharged.

**Table 2:** Laboratory findings in Case 2.

	Reference interval	ED admission	Time_6h_	Time_20h_ ICU admission	Time_68h_
**Paracetamol, μg/mL**	<5^a^	149.6	40.0	<5.0	–
**ALT, U/L**	<33	18	18	16	20
**Bilirubin, mg/dL**	<1.2	0.4	0.5	0.5	0.4
**PT (ratio)**	0.9–1.1	1.11	1.35	1.30	–
**APTT**	0.9–1.1	0.80	0.92	–	–
**Procalcitonin, ng/mL**	–	0.12	25.80	79.78	15.11
**CRP, mg/dL**	<0.5	<0.03	<0.03	<0.03	4.5

^a^Toxic concentrations after overdose: •4 h after overdose: >200. •8 h after overdose: 1056. •12 h after overdose: >50. ED, emergency department; ICU, intensive care unit; ALT, alanine aminotransferase; PT, prothrombin time; aPTT, activated partial thromboplastin time; CRP, C-reactive protein.

## Discussion

Procalcitonin is the most useful biomarker in the diagnosis of infection, prediction of bacteriemia, evaluation of infection severity, and decision-making regarding the start of antimicrobial therapy. In addition, this marker is used to evaluate response to antimicrobial therapy for progressive down-titration [3]. However, there are reports of elevated procalcitonin concentrations in patients with conditions other than infection [3], including paracetamol intoxication [5], which limits its suitability as an infection marker in these patients. However, procalcitonin is useful for assessing infection in patients with acute liver failure unrelated to paracetamol intoxication [6].

The mechanism by which paracetamol causes an increase in procalcitonin concentrations is still unclear. We report a case series of two patients with procalcitonin elevation related to paracetamol intoxication that affected liver function differently, as determined based on liver function markers. These markers were not elevated in Case 2 either at admission or during hospital stay.

Tschiedel et al. [7] recently described two possible mechanisms to explain the increase in procalcitonin levels in a setting of paracetamol intoxication. The first mechanism is the hepatotoxicity induced by the medication, which may trigger the production of specific cytokines such as interleukin 2 or tumor necrosis factor alpha, which mediate the elevation of procalcitonin concentrations. Another mechanism may involve other tissues such as the endothelium. In the same line, Lovas et al. [4], in a case of paracetamol intoxication, suggest the involvement of other damaged tissues, including immune cells, in the increase of procalcitonin.

The agent that causes paracetamol-induced hepatotoxicity is metabolite N-acetyl-*p*-benzoquinonimine (NAPQI), which is produced by the cythorchrome P450 system. An experimental study in rats suggested that the activity of these enzymes could be induced by the ingestion of paracetamol at non-hepatotoxic doses, and some degree of hepatotoxicity can be developed without an elevation of aminotransferases [9]. Tschiedel et al. [7] suggested the involvement of other organs such as endothelial cells in the increase of paracetamol-induced procalcitonin elevation through the production of NAPQI.

Finally, Ahn et al. [5] recently proposed that staggered overdose ingestion, which could not be confirmed in Case 2, could generate a different pattern of hepatotoxicity that does not cause the elevation of liver function markers, as compared to single-dose ingestion. These authors suggest that an analysis of procalcitonin subtypes in patients with paracetamol intoxication would contribute to better understand the mediators of the secretion of this marker. However, subtypes of procalcitonin cannot be identified with the technologies currently available.

## Conclusions

We report two cases of procalcitonin elevation resulting from paracetamol overdose that occurred independently from levels of liver function markers and without evidence of infection. Further studies are needed to better understand the mechanism of procalcitonin elevation in this setting. Nevertheless, clinicians should be aware of the limitations of using procalcitonin as a marker for the detection and follow-up of infection in patients with paracetamol overdose.
